# 2,6,7-Trioxa-1-phosphabicyclo­[2.2.2]octan-4-ylmethanol 1-sulfide

**DOI:** 10.1107/S1600536808041937

**Published:** 2008-12-17

**Authors:** Qi Yang, Ye-qin Zhang, Jian-qian Huang, Jian Men, Guo-wei Gao

**Affiliations:** a College of Polymer Science and Engineering, Sichuan University, Chengdu 610065, People’s Republic of China; b College of Chemistry, Sichuan University, Chengdu 610064, People’s Republic of China

## Abstract

The title compound, C_5_H_9_O_4_PS, was synthesized by the reaction of penta­erythritol with thio­phosphoryl chloride. In the crystal structure, the three six-membered rings all adopt boat conformations. Mol­ecules form chains along the *c* axis *via* inter­molecular O—H⋯O hydrogen bonds.

## Related literature

For a general background to the synthesis and applications of the title compound, see: Bourbigot & Duquesne (2007 ▶); Fontaine *et al.* (2008 ▶); Le Bras *et al.* (1997 ▶); Ratz & Aweeting (1964 ▶).
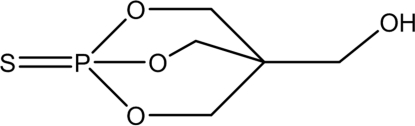


## Experimental

### 

#### Crystal data

C_5_H_9_O_4_PS
*M* *_r_* = 196.16
Orthorhombic, 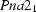

*a* = 11.571 (3) Å
*b* = 9.724 (3) Å
*c* = 7.112 (4) Å
*V* = 800.2 (6) Å^3^
*Z* = 4
Mo *K*α radiation
μ = 0.57 mm^−1^
*T* = 292 (2) K
0.44 × 0.40 × 0.24 mm

#### Data collection

Enraf–Nonius CAD-4 diffractometer
Absorption correction: for a sphere (Farrugia, 1999 ▶) *T*_min_ = 0.861, *T*_max_ = 0.863
1224 measured reflections
949 independent reflections
864 reflections with > 2s(*I*)
*R*_int_ = 0.009
3 standard reflections every 80 reflections intensity decay: 0.3%

#### Refinement

*R*[*F* ^2^ > 2σ(*F* ^2^)] = 0.037
*wR*(*F* ^2^) = 0.106
*S* = 1.04
949 reflections
101 parameters
1 restraint
H-atom parameters constrained
Δρ_max_ = 0.45 e Å^−3^
Δρ_min_ = −0.23 e Å^−3^
Absolute structure: Flack (1983 ▶), 137 Friedel pairs
Flack parameter: −0.04 (19)

### 

Data collection: *DIFRAC* (Gabe *et al.*, 1993 ▶); cell refinement: *DIFRAC*; data reduction: *NRCVAX* (Gabe *et al.*, 1989 ▶); program(s) used to solve structure: *SHELXS97* (Sheldrick, 2008 ▶); program(s) used to refine structure: *SHELXL97* (Sheldrick, 2008 ▶); molecular graphics: *ORTEP-3* (Farrugia, 1997 ▶); software used to prepare material for publication: *SHELXL97*.

## Supplementary Material

Crystal structure: contains datablocks global, I. DOI: 10.1107/S1600536808041937/rk2119sup1.cif

Structure factors: contains datablocks I. DOI: 10.1107/S1600536808041937/rk2119Isup2.hkl

Additional supplementary materials:  crystallographic information; 3D view; checkCIF report

## Figures and Tables

**Table 1:** Hydrogen-bond geometry (Å, °)

*D*—H⋯*A*	*D*—H	H⋯*A*	*D*⋯*A*	*D*—H⋯*A*
O4—H4⋯O2^i^	0.82	2.20	2.886 (6)	141

Symmetry code: (i) 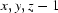.

**Fig. 1.**
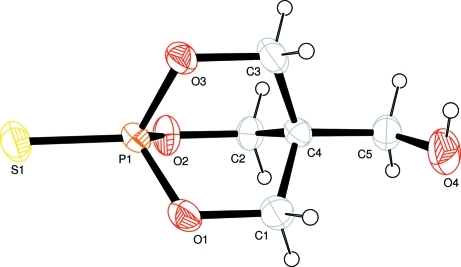
The asymmetric unit of title compound with the atom numbering scheme. Displacement ellipsoids are drawn at the 30% probability level. H atoms are presented as a small spheres of arbitrary radius.

## supplementary crystallographic information

### Comment

Intumescent flame retardant systems appear as an attractive topic and represent a wide and interesting area of research (Bourbigot & Duquesne, 2007). The use of pentaerythritol (Le Bras *et al.*, 1997) as char former in intumescent formulations which composed of three components, i.e. an acid source, a char forming agent and a blowing agent for thermoplastics is associated with migration, water solubility and other problems. Those problems were solved by synthesis of additives that concentrate the three intumescent flame retardant elements in one molecule (Fontaine *et al.*, 2008). The compound synthesized (Ratz & Aweeting, 1964) which has little intumescence is the intermediate product of the concentrate intumescent flame retardant.

In the molecule of the title compound (Fig.1), three six–membered rings adopt boat conformations. The bond angle of C3—C4—C5 is 112.7 (4)° which is bigger than one of *sp*^3^ hybrid, it may be the result of the co–existence of the three six–membered rings attached at C5. The torsion angles of S1/P1/O3/C3 and O1/C1/C4/C5 are -178.7 (3)° and -178.4 (4)°, respectively. Intermolecular O—H···O hydrogen bonds link the molecules with formation chains along *c* axis and effective stabilized the crystal structure (Table).

### Experimental

A mixture of 62.6 g (0.46 mol) pentaerythritol and 77.9 g (0.46 mol) thiophosphoryl chloride was heated at 418 K in a 250 ml round–bottomed flask equipped for reflux, protected from atmospheric moisture and equipped with magnetic stirrer. Evolution of hydrogen chloride ceased after 5 h. The resulting cake was extracted with 150 ml boiling water and cooled to room temperature. During the extracting some material remained undissolved and collected as a heavy oil at the bottom of the flask. The aqueous solvent was separated from this oil by decantation through a folded filter. The product was crystallized from water and afforded white crystals (62 g, yield 68.7%, m.p. 431–433 K).

### Refinement

H atoms were positioned geometrically (C—H = 0.97Å and O—H = 0.82 Å) and refined using a riding model, with *U*_iso_(H) = 1.2*U*_eq_(C), *U*_iso_(H) = 1.5*U*_eq_(O).

### Crystal data

**Table:**

C_5_H_9_O_4_PS	*F*(000) = 408
*M**_r_* = 196.16	*D*_x_ = 1.628 Mg m^−^^3^
Orthorhombic, *P**n**a*2_1_	Mo *K*α radiation, λ = 0.71073 Å
Hall symbol: P 2c -2n	Cell parameters from 16 reflections
*a* = 11.571 (3) Å	θ = 4.5–7.2°
*b* = 9.724 (3) Å	µ = 0.57 mm^−^^1^
*c* = 7.112 (4) Å	*T* = 292 K
*V* = 800.2 (6) Å^3^	Block, colourless
*Z* = 4	0.44 × 0.40 × 0.24 mm

### Data collection

**Table:**

Enraf–Nonius CAD-4 diffractometer	864 reflections with > 2s(*I*)
Radiation source: Fine–focus sealed tube	*R*_int_ = 0.009
Graphite	θ_max_ = 25.5°, θ_min_ = 2.7°
ω/2θ scans	*h* = −13→13
Absorption correction: for a sphere (Farrugia, 1999)	*k* = −11→11
*T*_min_ = 0.861, *T*_max_ = 0.863	*l* = −8→2
1224 measured reflections	3 standard reflections every 80 reflections
949 independent reflections	intensity decay: 0.3%

### Refinement

**Table:**

Refinement on *F*^2^	Secondary atom site location: Difmap
Least-squares matrix: Full	Hydrogen site location: Geom
*R*[*F*^2^ > 2σ(*F*^2^)] = 0.037	H-atom parameters constrained
*wR*(*F*^2^) = 0.106	*w* = 1/[σ^2^(*F*_o_^2^) + (0.0792*P*)^2^ + 0.1948*P*] where *P* = (*F*_o_^2^ + 2*F*_c_^2^)/3
*S* = 1.04	(Δ/σ)_max_ < 0.001
949 reflections	Δρ_max_ = 0.45 e Å^−^^3^
101 parameters	Δρ_min_ = −0.23 e Å^−^^3^
1 restraint	Absolute structure: Flack (1983), 137 Friedel pairs
Primary atom site location: Direct	Flack parameter: −0.04 (19)

### Special details

**Table:**

Geometry. All s.u.'s (except the s.u. in the dihedral angle between two l.s. planes) are estimated using the full covariance matrix. The cell s.u.'s are taken into account individually in the estimation of s.u.'s in distances, angles and torsion angles; correlations between s.u.'s in cell parameters are only used when they are defined by crystal symmetry. An approximate (isotropic) treatment of cell s.u.'s is used for estimating s.u.'s involving l.s. planes.
Refinement. Refinement of *F*^2^ against ALL reflections. The weighted *R*–factor *wR* and goodness of fit *S* are based on *F*^2^, conventional *R*–factors *R* are based on *F*, with *F* set to zero for negative *F*^2^. The threshold expression of *F*^2^ > 2σ(*F*^2^) is used only for calculating *R*–factors(gt) *etc*. and is not relevant to the choice of reflections for refinement. *R*–factors based on *F*^2^ are statistically about twice as large as those based on *F*, and *R*–factors based on ALL data will be even larger.

### Fractional atomic coordinates and isotropic or equivalent isotropic displacement parameters (Å^2^)

**Table:**

	*x*	*y*	*z*	*U*_iso_*/*U*_eq_	
S1	0.43219 (11)	0.07576 (14)	1.1866 (2)	0.0617 (4)	
P1	0.32384 (8)	0.09916 (9)	0.98909 (19)	0.0389 (3)	
O1	0.2853 (3)	−0.0370 (3)	0.8907 (5)	0.0534 (8)	
O2	0.2054 (3)	0.1676 (3)	1.0501 (5)	0.0520 (8)	
O3	0.3650 (2)	0.1924 (3)	0.8211 (5)	0.0509 (8)	
O4	0.1030 (4)	0.0757 (5)	0.3982 (6)	0.0812 (13)	
H4	0.1541	0.1153	0.3394	0.122*	
C3	0.2796 (4)	0.2118 (5)	0.6721 (8)	0.0575 (12)	
H3A	0.3115	0.1806	0.5534	0.069*	
H3B	0.2617	0.3089	0.6603	0.069*	
C1	0.1994 (4)	−0.0202 (5)	0.7394 (8)	0.0545 (11)	
H1A	0.1299	−0.0711	0.7703	0.065*	
H1B	0.2302	−0.0566	0.6227	0.065*	
C2	0.1224 (3)	0.1837 (5)	0.8992 (7)	0.0470 (10)	
H2A	0.1019	0.2801	0.8873	0.056*	
H2B	0.0527	0.1329	0.9296	0.056*	
C4	0.1702 (3)	0.1327 (4)	0.7147 (7)	0.0401 (9)	
C5	0.0792 (4)	0.1483 (5)	0.5622 (7)	0.0557 (12)	
H5A	0.0055	0.1173	0.6115	0.067*	
H5B	0.0716	0.2450	0.5314	0.067*	

### Atomic displacement parameters (Å^2^)

**Table:**

	*U*^11^	*U*^22^	*U*^33^	*U*^12^	*U*^13^	*U*^23^
S1	0.0617 (7)	0.0732 (7)	0.0503 (7)	0.0055 (5)	−0.0140 (6)	0.0133 (7)
P1	0.0419 (5)	0.0398 (4)	0.0351 (5)	0.0032 (4)	0.0019 (5)	0.0075 (5)
O1	0.072 (2)	0.0367 (14)	0.0515 (19)	0.0077 (13)	−0.0097 (18)	0.0070 (16)
O2	0.0443 (14)	0.077 (2)	0.0349 (15)	0.0123 (14)	0.0007 (14)	−0.0066 (16)
O3	0.0430 (14)	0.0607 (17)	0.049 (2)	−0.0084 (13)	−0.0033 (15)	0.0200 (17)
O4	0.101 (3)	0.106 (3)	0.037 (2)	−0.015 (2)	−0.003 (2)	0.001 (2)
C3	0.052 (2)	0.074 (3)	0.047 (3)	−0.003 (2)	−0.005 (3)	0.025 (3)
C1	0.069 (3)	0.045 (2)	0.048 (3)	0.0042 (19)	−0.004 (2)	−0.003 (2)
C2	0.042 (2)	0.056 (2)	0.043 (3)	0.0064 (17)	−0.003 (2)	−0.004 (2)
C4	0.0401 (19)	0.0421 (18)	0.038 (2)	0.0000 (15)	0.0020 (18)	0.0051 (19)
C5	0.057 (3)	0.065 (3)	0.045 (3)	−0.005 (2)	−0.008 (2)	0.001 (2)

### Geometric parameters (Å, °)

**Table:**

S1—P1	1.8966 (19)	C3—H3B	0.9700
P1—O1	1.562 (3)	C1—C4	1.535 (6)
P1—O3	1.573 (3)	C1—H1A	0.9700
P1—O2	1.583 (3)	C1—H1B	0.9700
O1—C1	1.474 (6)	C2—C4	1.508 (6)
O2—C2	1.449 (5)	C2—H2A	0.9700
O3—C3	1.461 (5)	C2—H2B	0.9700
O4—C5	1.390 (6)	C4—C5	1.519 (7)
O4—H4	0.8200	C5—H5A	0.9700
C3—C4	1.512 (6)	C5—H5B	0.9700
C3—H3A	0.9700		
			
O1—P1—O3	103.55 (19)	C4—C1—H1B	109.7
O1—P1—O2	103.38 (18)	H1A—C1—H1B	108.2
O3—P1—O2	103.18 (18)	O2—C2—C4	111.4 (3)
O1—P1—S1	114.77 (13)	O2—C2—H2A	109.3
O3—P1—S1	115.56 (13)	C4—C2—H2A	109.3
O2—P1—S1	114.78 (15)	O2—C2—H2B	109.3
C1—O1—P1	115.2 (2)	C4—C2—H2B	109.3
C2—O2—P1	114.6 (3)	H2A—C2—H2B	108.0
C3—O3—P1	114.9 (2)	C2—C4—C3	108.3 (4)
C5—O4—H4	109.5	C2—C4—C5	109.5 (3)
O3—C3—C4	110.8 (4)	C3—C4—C5	112.7 (4)
O3—C3—H3A	109.5	C2—C4—C1	107.5 (4)
C4—C3—H3A	109.5	C3—C4—C1	109.3 (3)
O3—C3—H3B	109.5	C5—C4—C1	109.3 (4)
C4—C3—H3B	109.5	O4—C5—C4	114.3 (4)
H3A—C3—H3B	108.1	O4—C5—H5A	108.7
O1—C1—C4	109.9 (4)	C4—C5—H5A	108.7
O1—C1—H1A	109.7	O4—C5—H5B	108.7
C4—C1—H1A	109.7	C4—C5—H5B	108.7
O1—C1—H1B	109.7	H5A—C5—H5B	107.6
			
O3—P1—O1—C1	−54.2 (3)	O2—C2—C4—C3	−58.1 (5)
O2—P1—O1—C1	53.1 (3)	O2—C2—C4—C5	178.5 (4)
S1—P1—O1—C1	178.9 (3)	O2—C2—C4—C1	59.9 (4)
O1—P1—O2—C2	−53.5 (3)	O3—C3—C4—C2	59.4 (5)
O3—P1—O2—C2	54.1 (3)	O3—C3—C4—C5	−179.2 (4)
S1—P1—O2—C2	−179.3 (3)	O3—C3—C4—C1	−57.4 (5)
O1—P1—O3—C3	54.9 (3)	O1—C1—C4—C2	−59.6 (4)
O2—P1—O3—C3	−52.6 (4)	O1—C1—C4—C3	57.7 (5)
S1—P1—O3—C3	−178.7 (3)	O1—C1—C4—C5	−178.4 (4)
P1—O3—C3—C4	−1.3 (5)	C2—C4—C5—O4	−165.6 (4)
P1—O1—C1—C4	0.8 (5)	C3—C4—C5—O4	73.8 (6)
P1—O2—C2—C4	−1.0 (5)	C1—C4—C5—O4	−48.1 (5)

### Hydrogen-bond geometry (Å, °)

**Table:**

*D*—H···*A*	*D*—H	H···*A*	*D*···*A*	*D*—H···*A*
O4—H4···O2^i^	0.82	2.20	2.886 (6)	141

Symmetry codes: (i) *x*, *y*, *z*−1.
